# *Lactobacillus gasseri* PA-3 Uses the Purines IMP, Inosine and Hypoxanthine and Reduces Their Absorption in Rats

**DOI:** 10.3390/microorganisms5010010

**Published:** 2017-03-08

**Authors:** Naruomi Yamada, Chizuru Saito-Iwamoto, Marie Nakamura, Misato Soeda, Yoshika Chiba, Hiroshi Kano, Yukio Asami

**Affiliations:** Food Science Research Laboratories, R & D Division, Meiji Co., Ltd., 540 Naruda, Odawara, Kanagawa 250-0862, Japan; chizuru.saitou@meiji.com (C.S.-I.); marie.nakamura@meiji.com (M.N.); misato.soeda.ba@meiji.com (M.S.); yoshika.chiba@meiji.com (Y.C.); hiroshi.kano@meiji.com (H.K.); yukio.asami@meiji.com (Y.A.)

**Keywords:** lactic acid bacteria, *Lactobacillus gasseri* PA-3, decreasing the absorption of purines

## Abstract

Excessive intake of purine-rich foods elevates serum levels of uric acid. Animal and fish meats contain high amounts of inosine and its related purines, and the reduction of taking those purines is crucial for the improvement of serum uric acid levels. We previously showed that *Lactobacillus gasseri* PA-3 (PA-3) incorporates adenosine and its related purines and that oral treatment with PA-3 reduced adenosine absorption in rats. This study investigated whether PA-3 also incorporates IMP (inosine 5′-monophosphate), inosine, and hypoxanthine, and whether it reduces their absorption in rats. PA-3 was incubated in vitro with radioisotope (RI)-labeled IMP, inosine, and hypoxanthine, and the incorporation of these compounds by PA-3 was evaluated. In addition, rats were orally administered PA-3 along with RI-labeled inosine 5′-monophosphate, inosine, or hypoxanthine, and the ability of PA-3 to attenuate the absorption of these purines was determined. PA-3 incorporated all three purines and displayed greater proliferation in the presence than in the absence of these purines. Oral administration of PA-3 to rats reduced the absorption of IMP, inosine, and hypoxanthine. These results indicate that PA-3 reduces the absorption of purines contained in foods and it is expected that PA-3 contributes attenuation of the excessive intake of dietary purines.

## 1. Introduction

Uric acid is the metabolic end product of purines in humans, and increased serum uric acid levels are known to cause gout and hyperuricemia [1,2]. The intake of purine-rich food has been correlated with elevated serum concentrations of uric acid. The nucleic acids of foodstuffs such as vegetables consist mainly of adenine and guanine, with these foodstuffs having little effect on the development of hyperuricemia. In contrast, meats from animals, fish, and some shrimp contain high amounts of hypoxanthine. High intake of these foods has been reported to be associated with elevated serum levels of uric acid, with the latter correlated with the risk of gout [3,4,5,6]. Elevated serum uric acid levels may be prevented by reducing the absorption of inosine and related purine compounds (e.g., hypoxanthine and inosine 5′-monophosphate (IMP)) rather than adenosine and its related compounds.

A strategy aimed at reducing the intestinal absorption of food-derived purines is to eat a purine-reduced diet. In Japan, guidelines for the management of hyperuricemia and gout include lifestyle interventions such as nutritional therapy, with a recommended intake of dietary purines of ≤400 mg/day [7]. However, continuous nutritional therapy is not always easy, owing to the reduced palatability of purine-reduced foods, as flavorful foods generally contain high levels of purines such as umami components [8]. Another strategy is to eat foods that reduce serum uric acid level in the same way as drugs such as allopurinol [9], which inhibit the activity of hepatic xanthine oxidase (XO). A third, possibly more efficient strategy is to eat combinations of foodstuffs that have different mechanisms of action in decreasing serum uric acid levels. For example, ingestion of foodstuffs that reduce the absorption of purines in the human intestine may reduce serum uric acid levels. Lactic acid bacteria, such as lactobacilli, produce lactic acid as a major metabolic end product of carbohydrate fermentation. Oral intake of *Lactobacillus* may have beneficial effects for the host, by, for example, activating immune responses. Although purines are thought to be taken up and incorporated by *Lactococcus* [10,11], few reports have investigated the effect of *Lactobacillus* ingestion on hyperuricemia. We hypothesized that lactic acid bacteria ingested with food may take up purines in hosts’ intestines, reducing purine absorption by the latter. *Lactobacillus gasseri* PA-3 (PA-3) was shown to degrade purine nucleosides to purine bases, which are not easily absorbed by intestinal cells as nucleosides [12]. PA-3 has also been shown to take up exogenous AMP (Adenosine 5′-monophosphate), adenosine, and adenine in vitro [13], and oral administration of PA-3 to rats, along with AMP or adenosine, reduced the absorption of these purines by rat intestines [12]. However, the ability of PA-3 to reduce the absorption of dietary inosine and its related compounds remains unclear. This study therefore investigated whether PA-3 takes up and incorporates hypoxanthine, inosine, and IMP in vitro and whether it reduces the absorption of these purines in rats in vivo.

## 2. Experimental Section

### 2.1. Reagents, Radioactive Compounds, Media, and Lactic Acid Bacteria

Inosine 5′-monophosphate (IMP), inosine, and hypoxanthine were purchased from Wako (Osaka, Japan) and used without further purification. All other reagents were of commercially available grade. (8-^14^C) Inosine 5′-monophosphate diammonium salt (^14^C-IMP) (50.0 mCi/mmol) and (8-^14^C) inosine (^14^C-inosine) (53.0 mCi/mmol) were purchased from Moravek Biochemicals (La Brea, CA, USA), and (8-^14^C) hypoxanthine (^14^C-hypoxanthine) (55.0 mCi/mmol) was obtained from American Radiolabeled Chemicals (St. Louis, MO, USA). OptiPhase Super Mix, a liquid scintillation cocktail, was obtained from Perkin Elmer (Waltham, MA, USA). Table 1 shows the composition of the defined growth medium containing the pyrimidines thymine, cytidine and 2′-deoxyuridine (DM-py), a medium slightly modified from that previously reported [14]. *Lactobacillus gasseri* PA-3 (PA-3) was isolated by Meiji Co., Ltd., Tokyo, Japan. *Lactobacillus gasseri* ATCC 33323 (ATCC 33323) was obtained from RIKEN (Saitama, Japan). These strains were propagated in MRS broth (DIFCO, Detroit, MI, USA) at 37 °C under anaerobic conditions (AnaeroPack-Anaero, MGC, Tokyo, Japan). Frozen cultures were sub-cultured twice for 20 h each prior to assays.

### 2.2. Assays of Purine Nucleoside and Nucleotide Degradation

Each 100 μL standard assay mixture for purine nucleoside degradation contained 0.02 μmol inosine, 10 μmol sodium phosphate buffer (pH 7.0), and OD_650_ = 5 of PA-3, whereas each 100 μL standard assay mixture for purine nucleotide degradation contained 0.02 μmol IMP, 0.5 μmol magnesium chloride, 2.5 μmol Tris-HCl buffer (pH 7.5), and OD_650_ = 5 of PA-3. The mixtures for both assays were incubated for 60 min at 37 °C and the reactions terminated by adding 100 μL 5% (*v*/*v*) trifluoroacetic acid (TFA). The mixtures were centrifuged at 4000× *g* for 10 min, and the supernatants were filtered through 0.22 μm filters.

### 2.3. Measurement of Purines by HPLC

Nucleoside or nucleotide degradation in the filtrate samples were analyzed by high pressure liquid chromatography (HPLC). Reductions in substrate (IMP or inosine) concentrations and increases in product (inosine or hypoxanthine) concentrations were analyzed on a 600E HPLC separation module (Waters Corporation, Milford, MA) equipped with a Develosil RPAQUEOUS-AR-3 column (150 nm × 2 mm i.d.; Nomura Chemical, Aichi, Japan) at a flow rate of 0.2 mL/min at 40 °C. Following elution with Solvent A (20 mM sodium phosphate, pH 6.0), the column was eluted with a linear gradient of 0%–20% Solvent B (50% acetonitrile, 20 mM sodium phosphate, pH 6.0) in 15 min, with the eluent monitored at 254 nm. One unit of enzyme was defined as the amount of enzyme catalyzing the formation of inosine or hypoxanthine, at a rate of 1 pmol/min per 1.0 × 10^9^ cells, under the assay conditions described above.

### 2.4. Uptake of Purines in DM-py

PA-3 was cultured in DM–py (3.0 × 10^7^ cells/mL) in the presence of ^14^C-IMP, ^14^C-inosine, or ^14^C-hypoxanthine (0.2 μCi/mL each) for 0, 30, 60, or 120 min at 37 °C under anaerobic conditions. Equal amounts of 5% (*v*/*v*) TFA were added, and the bacterial cells were collected by filtration (0.22 μm) and washed three times with 10 mL saline. The radioactivity of harvested bacterial cells in a scintillation cocktail (OptiPhase SuperMix, Perkin Elmer, Waltham, MA, USA) was measured by a liquid scintillation analyzer (Tri-Carb 3110TR, Perkin Elmer, Waltham, MA, USA).

### 2.5. Proliferation Assay

DM-py containing IMP, inosine, or hypoxanthine was inoculated with 4.0 × 10^7^ PA-3 cells/mL. The final concentrations of IMP, inosine, and hypoxanthine were 400 μM, with no purine added to the control. The cells were cultured for 0, 4, or 6 h at 37 °C under anaerobic conditions, and OD_650_ was measured using a Bio Spec-1600 spectrophotometer (Shimadzu, Kyoto, Japan).

### 2.6. Viable Cell Counts

Cells were incubated anaerobically for 72 h at 37 °C on BCP plates (Eiken Chemical, Tokyo, Japan), and the number of viable cells was determined by the dilution method.

### 2.7. Animal Experiments

The animal protocols used in this work were evaluated and approved by the Ethical Committee for Animal Experiments of Meiji Co., Ltd., Tokyo, Japan (2014_3871_0303, 2015_3871_0048, and 2015_3871_0159), and all animal studies were performed according to the guidelines of the Ethical Committee for Animal Experiments of Meiji Co., Ltd.

Male Wistar rats, aged 8 weeks and weighing 190–210 g, were purchased from SLC (Shizuoka, Japan) and housed in an animal laboratory under controlled ambient conditions, including a temperature of 20–26 °C, relative humidity of 40%–70%, and a 12 h/12 h light/dark cycle. All rats had free access to a normal diet, the CRF-1 laboratory diet (Oriental Yest Co., Tokyo, Japan), and tap water ad libitum for one week. Based on body weight stratification, they were divided randomly into three groups, a control group (*n* = 4), a purine group (*n* = 5), and a purine-PA-3 group (*n* = 5), and fasted for 16 h. Control rats were orally administrated 2 mL saline by gastric gavage; rats in the purine group were orally administrated 10 μCi ^14^C-IMP, ^14^C-inosine or ^14^C-hypoxanthine in 2 mL; and rats in the purine-PA-3 group were orally administrated 10 μCi ^14^C-IMP, ^14^C-inosine or ^14^C-hypoxanthine plus 1.0 × 10^10^ PA-3 cells in 2 mL. Blood samples (60 μL) were collected at 0, 15, 30, 45, 60, 90, 120, and 180 min and mixed with an equal volume of 2 mg/mL Na_2_-EDTA. The samples were added to a scintillation cocktail (OptiPhase SuperMix, Perkin Elmer) and radioactivity was measured in a liquid scintillation counter (LSC-6000, Hitachi Aloka Medical, Tokyo, Japan).

### 2.8. Statistical Analyses

Groups were compared statistically by *t*-tests, with *p* < 0.05 defined at statistically significant.

## 3. Results

### 3.1. Degradation of Purines by ATCC 33323 and PA-3

**Figure 1:**
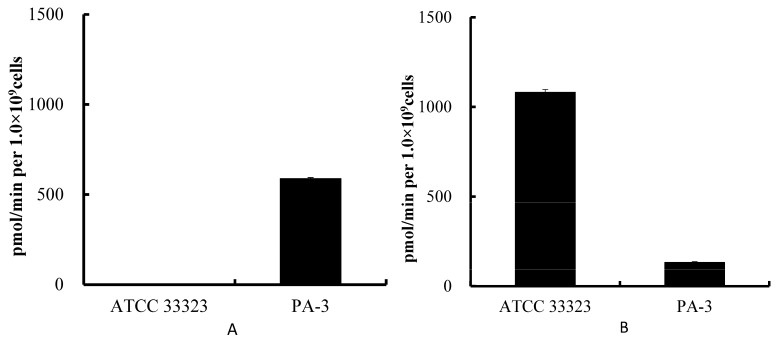
Enzymatic conversion of inosine to hypoxanthine (**A**) and Inosine 5′-monophosphate (IMP) to inosine (**B**) by *Lactobacillus gasseri* ATCC 33323 and PA-3 in vitro. Each value represents the mean ± SD of three samples.

We compared the ability of PA-3 and a type strain of *L. gasseri*, ATCC33323, to convert IMP to inosine and inosine to hypoxanthine. PA-3 displayed a significantly higher ability to convert inosine to hypoxanthine (Figure 1A), but a significantly lower ability to degrade IMP (Figure 1B), than ATCC 33323.

### 3.2. Uptake of Purines

**Figure 2:**
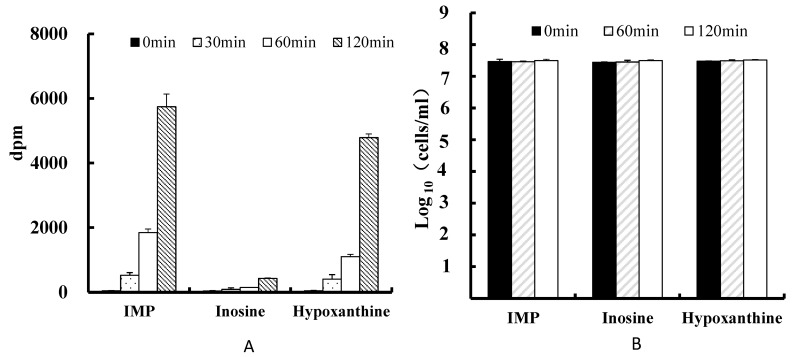
(**A**) Uptake of ^14^C-IMP, ^14^C-inosine, and ^14^C-hypoxanthine in DM-py by 3 × 10^7^ *Lactobacillus gasseri* PA-3 cells/mL after 0, 30, 60, and 120 min. (**B**) Numbers of viable PA-3 cells after culture in DM-py containing IMP (inosine 5′-monophosphate), inosine, or hypoxanthine. Values represent the means ± SD of three samples.

PA-3 incorporated each of the purines tested, ^14^C-IMP, ^14^C-inosine, and ^14^C-hypoxanthine, with ^14^C-inosine showing the lowest uptake of the three (Figure 2).

### 3.3. Proliferation Assay

**Figure 3:**
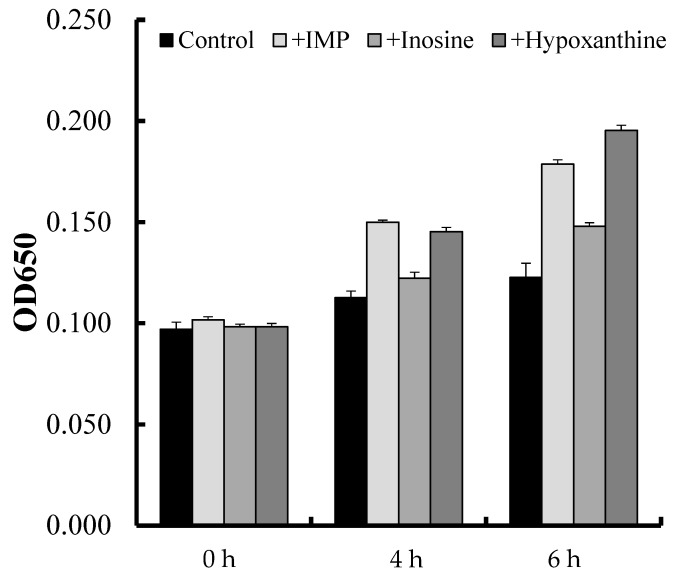
PA-3 proliferation in the presence of IMP, inosine, or hypoxanthine for 0, 4, or 6 h. Values represent means ± SD of three samples.

PA-3 showed greater proliferation when cultured in the presence than in the absence of IMP, inosine, or hypoxanthine (Figure 3). PA-3 proliferation was lower in the presence of inosine than in the presence of IMP or hypoxanthine (Figure 3).

### 3.4. Animal Experiment

**Figure 4:**
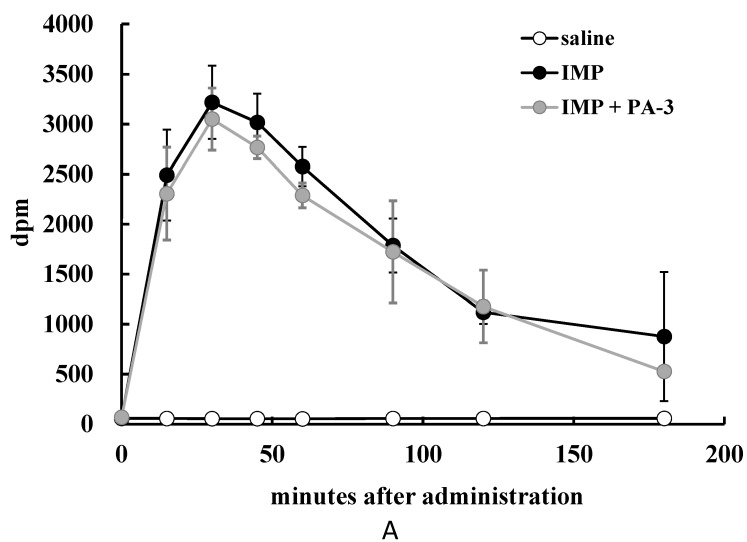

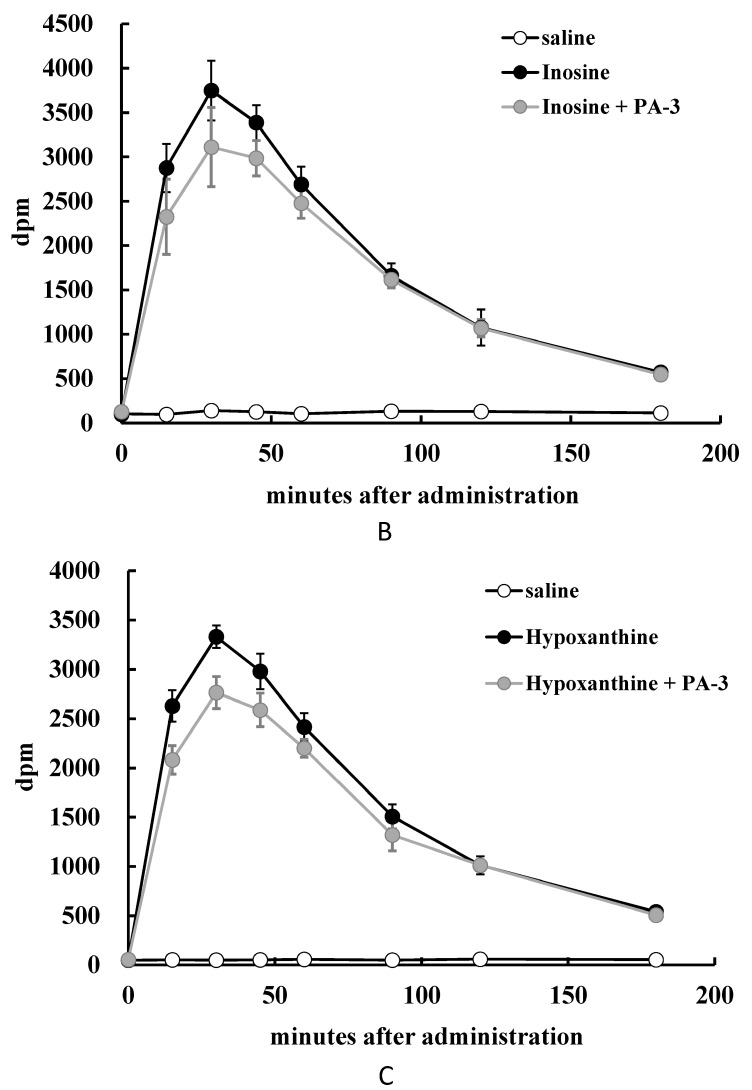
Effect of PA-3 on purine absorption in rats. Rats were orally administered PA-3 and (**A**) ^14^C-IMP; (**B**) ^14^C-inosine; or (**C**) ^14^C-hypoxanthine, and blood samples were collected at 0, 15, 30, 45, 60, 90, 120, and 180 min. The radioactivity of blood samples was measured by a liquid scintillation counter. Values represent means ± SD of 4–5 rats. # *p* < 0.05, ## *p* < 0.01 compared with rats administered ^14^C-labelled purines alone.

Radioactivity was significantly lower in the blood of rats 60 min after administration of ^14^C-IMP plus PA-3 than after administration of ^14^C-IMP alone (Figure 4A). Similarly, the blood of rats administered ^14^C-inosine plus PA-3 showed significantly lower radioactivity after 15, 30, and 45 min than the blood of rats administered ^14^C-inosine alone (Figure 4B), and the blood of rats administered ^14^C-hypoxanthine plus PA-3 showed significantly lower radioactivity after at 15, 30, 45, 60, and 90 min than the blood of rats administered ^14^C-hypoxanthine alone (Figure 4C).

## 4. Discussion

Animal and fish meats are rich in inosine and related purine compounds, with high intake of these foods correlating with an increased risk of gout [3]. *Lactobacillus gasseri* has been shown to incorporate adenosine and related purines and to reduce their absorption in rat intestines [12]. This study investigated whether PA-3 also incorporated inosine and related purines and decreased the absorption of these purines in rat intestines.

We confirmed that PA-3 incorporated IMP, inosine, and hypoxanthine in vitro and showed greater proliferation in their presence than in their absence. Microorganisms, including lactic acid bacteria, synthesize AMP, and GMP (Guanosine 5′-monophosphate) from IMP, utilize ATP (Adenosine 5′-triphosphate) and GTP (Guanosine 5′-triphosphate) to synthesize DNA and RNA [10,11]. We found that genes in PA-3 encode enzymes that catalyze the conversion of IMP to AMP and GMP (unpublished data), suggesting that this bacterium can convert these compounds into ATP and GTP, respectively, which are incorporated into DNA and RNA. ATP is the general energy supplier in lactic acid bacteria, and high concentrations of ATPase are present in these bacterial cells [10]. These findings indicate that ATP plays an important role in the growth of lactic acid bacteria, including PA-3, and that PA-3 uses these purines in the synthesis of DNA and RNA, resulting in cell proliferation. This hypothesis can be confirmed by showing that radioactively-labeled purines taken up by PA-3 have been incorporated into the DNA and RNA fractions of cell lysates and are present as free ATP.

PA-3 was originally selected for its ability to convert purine nucleosides, such as inosine, to purine bases, such as hypoxanthine [12]. This study found that PA-3 uptake of hypoxanthine was greater than that of inosine, similar to findings with adenine and adenosine [13]. Some lactobacilli possess purine salvage enzymes, such as hypoxanthine-guanine phosphoribosyl transferase (HGPRT), which converts hypoxanthine to IMP [10,15]. Our findings suggest that PA-3 prefers to use purine bases rather than purine nucleosides for synthesis of nucleic acids through the salvage pathway. PA-3 incorporated nucleotides of both AMP [12,13] and IMP. PA-3 is believed to incorporate nucleotides via two pathways, one through a transporter on the cell surface and the other by incorporating nucleosides derived from nucleotides. Because PA-3 has little ability to convert nucleotides to nucleosides, this bacterium is unlikely to incorporate nucleosides derived from the degradation of nucleotides. Although PA-3 may incorporate nucleotides directly, no report to date has shown the uptake of purine nucleotides by lactic acid bacteria [10,16,17,18]. Further studies are required to show that PA-3 incorporates nucleotides without conversion to nucleosides.

This study also showed that PA-3 reduced the absorption of inosine and its related compounds by rat intestines. Because PA-3 incorporates low amounts of inosine, this bacterium may reduce rat absorption of IMP by direct uptake of this compound. Further studies are needed to clarify the mechanisms by which PA-3 takes up purine nucleotides. Results observed with inosine were likely due to its incorporation by PA-3 and subsequent degradation to hypoxanthine, which is less absorbed than inosine [19,20]. Results observed with hypoxanthine were likely due its uptake by PA-3, along with its low level absorption by the intestines. Thus, degradation of nucleosides and uptake of purines by PA-3 result in reduced absorption of purines by the intestines. Moreover, the effectiveness of nucleotides and nucleosides may be diminished because they were easily absorbed by the intestines.

This study demonstrated that PA-3 attenuated the intestinal uptake of inosines and related purines, as well as adenosines and related compounds. Reducing the intestinal absorption of food-derived purines is important to maintain normal serum uric acid levels. Although further studies are needed to analyze its mechanisms of action, PA-3 likely contributes to the health of individuals with high serum uric acid levels and hyperuricemia.

## 5. Conclusions

*Lactobacillus gasseri* PA-3 takes up and incorporates inosine and related compounds in vitro. Moreover, oral administration of PA-3 with the purines IMP, inosine, and hypoxanthine reduced the intestinal absorption of these purines in vivo. PA-3 may therefore protect individuals against elevated serum uric acid levels and resulting hyperuricemia and gout.

## Figures and Tables

**Table 1:** Composition of the defined growth medium with pyrimidines (thymine, cytidine and 2′-deoxyuridine) (DM-py).

Constituent	Final Concentration(/L)
D(+) Glucose	10 g
Potassium hydrogen phosphate	3.1 g
di-ammonium hydrogen citrate	2 g
Potassium dihydrogen phosphate	1.5 g
Potassium acetate	10 g
Calcium lactate 5H_2_O	1 g
Tween-80	1 g
Heptahydrated magnesium	500 mg
Sodium chloride	20 mg
Hydrated manganese sulphate	20 mg
Cobalt sulphate 7H_2_O	500 mg
Biotin	1 mg
Cyanocobalamin	0.02 mg
Folic acid	0.2 mg
para-aminobenzoic acid	0.2 mg
Nicotinic acid	10 mg
Calcium pantothenate	10 mg
Riboflavin	10 mg
Pyridoxal HCl	10 mg
Myo-inositol	10 mg
Ascorbic acid	0.5 mg
dl-aminobutyric acid	100 mg
l-phenylalanine	100 mg
l-serine	100 mg
l-threonine	100 mg
l-cycteine Hydrochloride Mnohydrate	100 mg
l-asparagine Mnohydrate	100 mg
l-isoleucine	100 mg
l-tyrosine	100 mg
l-tryptophan	100 mg
l-valine	100 mg
dl-alanine	200 mg
glycine	200 mg
l-histidine Hydrochloride Mnohydrate	200 mg
l-lysine Hydrochloride	200 mg
l-proline	200 mg
l-arginine	200 mg
l-leocine	200 mg
l-aspartic acid	300 mg
l-glutamic acid	300 mg
thymine	100 mg
cytidine	100 mg
2′-deoxyuridine	100 mg
